# Mammographic density and breast cancer pathological subtypes by menopausal status and body mass index

**DOI:** 10.1186/s13058-025-02142-2

**Published:** 2025-10-24

**Authors:** Julia Fernández-Morata, Marina Pollán, Nerea Fernández de Larrea-Baz, Vanessa Pachón-Olmos, Javier García-Pérez, Adela Castelló, María Ángeles Sierra, Pilar Lucas, Rafael Llobet, Agostina Stradella, Blanca Cantos, Teresa Ramón y Cajal, Marta Santisteban, Miguel Ángel Seguí, Ana Santaballa Bertrán, Mónica Granja, Julia Camps-Herrero, Sabela Recalde, Beatriz Nuñez-García, Nuria Calvo Verges, Beatriz Pérez-Gómez, Roberto Pastor-Barriuso, Virginia Lope

**Affiliations:** 1 Programa de Doctorado en Ciencias Biomédicas y Salud Pública, Instituto Mixto de Investigación Escuela Nacional de Sanidad UNED-IMIENS, Madrid, Spain; 2 Cancer and Environmental Epidemiology Unit, Department of Epidemiology of Chronic Diseases, National Center for Epidemiology, Instituto de Salud Carlos III (ISCIII), Madrid, Spain; 3 Consortium for Biomedical Research in Epidemiology and Public Health (CIBERESP), Madrid, Spain; 4 Servicio de Medicina Preventiva, Hospital Universitario La Paz-Carlos III-Cantoblanco, Madrid, Spain; 5 Institute of Computer Technology, Universitat Politècnica de València, Valencia, Spain; 6 Department of Medical Oncology, Multidisciplinary Breast Cancer Unit, Institut Català d’Oncologia, Idibell, Barcelona Spain; 7 Department of Medical Oncology, Hospital Universitario Puerta de Hierro, Majadahonda, Madrid Spain; 8 Department of Medical Oncology, Hospital de La Santa Creu I Sant Pau, Barcelona, Spain; 9 Department of Medical Oncology, Breast Cancer Unit, Clínica Universidad de Navarra, Pamplona, Spain; 10 IdiSNA, Navarra Institute for Health Research, Pamplona, Spain; 11 Department of Medical Oncology, Consorci Corporació Sanitaria Parc Tauli. Institut d’Investigació i Innovació Parc Taulí (I3PT-CERCA), Universitat Autònoma de Barcelona, Barcelona, Spain; 12 Medical Oncology Department, La Fe Health Research Institute (IISLaFe), La Fe University Hospital, Valencia, Spain; 13 Department of Medical Oncology, Hospital Universitario Clínico San Carlos, Madrid, Spain; 14 Radiology at Breast Health, Ribera Salud Hospitals, Valencia, Spain; 15 Department of Medical Oncology, Multidisciplinary Breast Cancer Unit, Institut Català d’Oncologia-Hospitalet, Barcelona, Spain

**Keywords:** Breast density, Breast neoplasm, Molecular subtypes, Body mass index, Menopausal status

## Abstract

**Background:**

Mammographic density (MD) is an established biomarker of breast cancer (BC) risk. However, its relationship to BC pathological subtypes remains unclear. This study aimed to investigate this association and assess whether it differs by body mass index (BMI) and menopausal status.

**Methods:**

MD percentage was assessed in the diagnostic mammograms of the contralateral breast of 714 BC patients recruited from eight Spanish hospitals. Participants completed an epidemiological questionnaire, and hospital researchers collected clinical and pathological data. Standardized prevalences (SPs) and standardized prevalence ratios (SPRs) for each BC pathological subtype across MD categories were estimated based on multinomial logistic regression models, both overall and stratified by BMI and menopausal status.

**Results:**

Mean MD was 26.1% (SD = 17.3). Although no statistically significant differences were detected, women with MD ≥ 50% had a 13% lower SP of hormone receptor positive tumors (SPR = 0.87; 95% CI 0.67–1.13), a 36% higher SP of human epidermal growth factor receptor 2 positive (HER2+) tumors (SPR = 1.36; 95% CI 0.72–2.58), and a 23% higher SP of triple negative (TN) tumors (SPR = 1.23; 95% CI 0.47–3.22), compared to those with MD < 10%. These patterns were mainly observed in pre/perimenopausal women and in those with BMI ≥ 25 kg/m^2^.

**Conclusions:**

High MD might be mainly associated with the development of more aggressive and non-hormone-dependent cancers, such as HER2+ and TN BC, especially among pre/perimenopausal an overweight women.

## Introduction

Breast cancer (BC) is the most commonly diagnosed female tumor worldwide and in Europe [1]. In Spain, it is also the tumor with the highest incidence (representing 30.3% of all incident cases) and the leading cause of cancer death (14.7% of total cancer deaths) in women in 2022 [2]. As of December 2020, female BC had become the most prevalent malignancy in Spain, with 516,827 reported cases [3]. Available treatments have contributed to high survival rates, but their use may entail long-term negative effects on health-related quality of life, highlighting the importance of prevention. Both primary and secondary prevention measures contribute to reducing the burden of BC, either avoiding its onset or reducing treatment-associated toxicity, as early-stage tumors often require less aggressive treatments. Although Spain performs well compared to other EU countries in terms of prevalence of some modifiable risk factors and screening implementation, there is still room for improvement. Smoking and alcohol consumption rates, as well as the prevalence of overweight, remain high, which has led to the strengthening of anti-smoking policies, increased taxes on alcohol and sugary drinks, and the development of a national strategic plan to reduce childhood obesity. Regarding secondary prevention, BC screening programs cover all women aged 50 to 69 (and also younger and older women in some regions), with participation rates exceeding 70%. However, participation remains low among the most vulnerable groups, such as migrant women or those with low socioeconomic or educational levels [4].

Mammographic density (MD) percent, defined as the proportion of dense fibroglandular tissue relative to fatty tissue in the breast, is an independent risk factor for BC [5, 6]. Having extremely dense breast tissue is associated with an almost two-fold increased risk of BC compared to having scattered dense breast tissue, after adjusting for age and body mass index (BMI) [7]. The population attributable risk associated with high MD appears to be higher in premenopausal (with estimates between 24 and 35%) than in postmenopausal women (between 13 and 17%) [8]. Although MD has a clear hereditary component, it can be influenced by several factors, being some of them modifiable. There is evidence that MD decreases progressively with age, with the transition to menopause, with parity, and with BMI. Conversely, the use of combined hormone therapy and older age at first birth have been associated with an increased MD. [9, 10].

There is increasing evidence indicating that BC subtypes—characterized by the presence or absence of specific receptors on tumor cells: hormone receptors (HR) (estrogen receptors (ER) and progesterone receptors (PR)), and human epidermal growth factor receptor 2 (HER2)—show etiological differences [11] and distinct biological behaviors and prognostic outcomes [12], which might be partially influenced by the characteristics of the breast tissue in which they arise. However, the association of MD with these subtypes remains unclear, since previous studies showed inconsistent results [13, 14]. A recent meta-analysis concluded that MD is a risk factor for most BC subtypes, and in particular, for HER2-positive tumours. Several studies included in this review found a positive association between MD and BC risk, regardless of tumor subtypes, while others reported evidence of heterogeneity across subtypes. [15]. These inconsistencies may reflect differences in study population, method of MD assessment, statistical power, subtype definitions, and covariates included in the models to control for potential confounding factors. Furthermore, previous studies have analysed the association between MD and molecular subtypes stratifying by other BC risk factors, such as BMI [16–18], age [19] or menopausal status [20], and found indications of possible effect modifications. Understanding these relationships could help tailor screening and intervention strategies in high-risk populations.

Of the 23 studies included in the meta-analysis by Bai et al., six were conducted in European populations, compared with 17 in American or Asian populations. In addition, 20 of them used the qualitative Breast Imaging-Reporting and Data System (BI-RADS) to measure MD, rather than quantitative methods [15]. In this context, the objective of the present study is to evaluate the relationship between MD percent and BC pathological subtypes, as well as to examine whether BMI and menopausal status modify this association, in a European population.

## Materials and methods

### Study population

The population of this study were participants in the Breast Cancer & Density Association Study (BCDAS), a multicenter case-case study. Recruitment was ambispective, including all women diagnosed with BC between January 2014 and March 2019 from the oncology departments of eight hospitals across four regions in Spain (Catalonia, Madrid, Valencian Region and Navarre). Patients were interviewed between October 2016 and December 2019, being 54% of them recruited retrospectively. Eligibility criteria included being over 18 years of age, having a confirmed histological diagnosis of BC (either invasive or in situ), and the ability to complete a telephone questionnaire administered by trained interviewers, including epidemiological and dietary information. Exclusion criteria included surgical interventions on the non-cancer breast before diagnosis with removal of breast tissue, breast reconstruction, breast augmentation, and the presence of a synchronous bilateral tumor. These patients were excluded due to lack of a baseline contralateral mammogram that could be used as “internal control”, and because they may represent a particular clinical/biological subgroup that could distort the results.

The BCDAS study protocol adhered to the principles of the Declaration of Helsinki and was formally approved by the Research Ethics and Animal Welfare Committee of the *Instituto de Salud Carlos III*. All participants signed an informed consent, and the database was pseudo-anonymized prior to conducting the statistical analyses.

### Mammographic density assessment

MD was determined on the craniocaudal digital mammogram of the contralateral (tumor-free) breast. Screening or diagnostic mammogram closest to the time of cancer diagnosis,, before starting any treatment, was collected. An experienced radiologist, blinded to clinical and pathological tumor information, estimated the percentage of MD assisted by DM-Scan, a free semi-automated computer tool that allows to estimate MD in 2-dimensional digital mammograms on a continuous scale and in DICOM format.

The development of DM-scan included a pre-processing stage to optimize mammograms before segmentation. First, contrast and brightness were normalized through histogram stretching to reduce variability and better reflect tissue density. Second, brightness was corrected to account for differences in breast thickness that may mimic dense tissue. Third, breast segmentation was performed, removing labels, background, or the pectoral muscle, with manual adjustments if needed. After these steps, a second threshold separated dense from fatty tissue. This allowed quantification of dense tissue (DT), fatty tissue (FT), and calculation of percentage density (PD = DT / (DT + FT) × 100). A previous study showed substantial agreement between MD estimates using DM-Scan and Cumulus, with concordance correlation coefficients over 0.80 [21].

### Covariates

Participants answered an epidemiological questionnaire that collected retrospective information, always referring to periods prior to the diagnosis of BC. The questionnaire included information on sociodemographic variables, family and personal medical history, hormonal and reproductive factors, lifetime tobacco and alcohol consumption, diet during the five years prior to diagnosis, and physical activity in the previous year.. Following recruitment, hospital researchers filled out a questionnaire containing clinical and pathological information. Tumor pathological subtypes were defined as HR positive tumors (HR+ : ER + and/or PR + with HER2-); HER2 positive tumors (HER2+ irrespective of ER or PR status); and triple negative tumors (TN: ER-, PR- and HER2-). ER, PR and HER2 positivity were defined according to ASCO/CAP guidelines [22–24].

### Statistical analyses

To evaluate significant differences among BC subtypes, the Pearson chi-square test was applied to categorical variables, whereas to test significant differences in MD means by categories of the variables, ANOVA F-test for heterogeneity was used, adjusted for age and BMI at diagnosis. A separate model was fitted for each variable. The prevalence of each BC pathological subtype by MD categories was standardized to the overall distribution of other sociodemographic, lifestyle and clinical characteristics. Percent MD was categorized according to the Boyd scale, but combining the 2 extreme categories (A + B and E + F): < 10% (reference), 10%– < 25% 25%– < 50% and ≥ 50%. A multinomial logistic regression model was fitted for this purpose, adjusted for age at diagnosis (< 50, 50–60, > 60 years), recruitment region, BMI at diagnosis (< 25, 25–30, ≥ 30 kg/m2), educational level (primary education or less, high school/vocational training, university), age at menarche (< 12, ≥ 12 years), age at first birth (< 20, 20–24, 25–29 or nulliparous, ≥ 30 years), menopausal status at diagnosis (pre/perimenopausal, postmenopausal), alcohol consumption in the year prior to diagnosis (no, yes), and breast biopsies prior to diagnosis with negative results (no, yes). These variables were selected based on both statistical and evidence-based criteria. Averages of the predicted probabilities of HR+ , HER2+ and TN tumors were then calculated (standardized prevalences, (SPs)) [25, 26]. These estimates can be interpreted as the prevalence of each pathological subtype that would have been observed in women with a particular characteristic if their distribution of all other characteristics had been the same as that of the whole sample of participants. This approach allowed controlling for confounding factors by direct standardization. Standardized prevalence ratios (SPRs) and 95% confidence intervals (95%CIs) of each BC subtype by MD category were also calculated, taking the lowest density category as the reference.

In addition, to explore potential heterogeneity by strata of BMI (< 25, ≥ 25 kg/m^2^) and menopausal status at diagnosis, we included interactions of these covariates with MD, grouped into two categories (< 25, ≥ 25%), in the corresponding multinomial logistic regression models. The Wald test was used for log-transformed SPRs. Finally, SPR and its 95%CI of each BC pathological subtype as a smooth function of MD was obtained from a multinomial logistic regression model based on restricted cubic splines for MD, with two internal knots at the 35th and 65th percentiles and boundary knots at the 5th and 95th percentiles. Statistical analyses were performed in Stata, version 18 (StataCorp) and graphics were generated in R, version 4 (R Foundation for Statistical Computing).

## Results

Of the initial sample of 1021 women with BC, 60 participants were deemed ineligible, 19 refused to participate, and 228 were excluded due to missing information on MD (156 women) or BC pathological subtype (72 women). Thus, the final study sample consisted of 714 women with BC. The sociodemographic and clinical characteristics at diagnosis, overall and by BC pathological subtype, are illustrated in Table 1. A total of 490 patients (68.6%) were diagnosed with HR+ BC, 139 (19.5%) with HER2+ , and 85 (11.9%) with TN tumors. The interviews were conducted on average one year after diagnosis (median = 363 days, range 0–5 years). Mean age at diagnosis was 56.8 years (range 22–94 years), and average BMI was 26.2 kg/m^2^. University education was completed by 34.0% of patients, and 62.0% were postmenopausal. Regarding differences among the three histological subtypes, women with TN tumors had lower frequency of regular alcohol consumption and were less likely to have their first child after the age of 30. Overall, mean MD was 26.1% (SD = 17.3). Younger women, those who were pre/perimenopausal at diagnosis, those with university education, with a lower BMI, with an older age at menarche or at first child, and participants with previous breast biopsies showed higher MD.

**Table 1:** Characteristics at diagnosis of breast cancer patients, overall and by pathological tumor subtype and mammographic density

		Pathologic tumor subtypes				Mammographic density	
	Total	HR+	HER2+	TN			
	n (%)	n (%)	n (%)	n (%)	*p*-value	mean (SD)	*p*-value^a^
*Total*	714 (100.0)	490 (68.6)	139 (19.5)	85 (11.9)			
Mammographic density (mean, SD)	26.1 (17.3)	25.5 (17.1)	27.1 (17.7)	27.4 (17.5)	0.467		
Age^b^							
22–49	237 (33.2)	164 (33.5)	42 (30.2)	31 (36.9)	0.129	38.7 (18.0)	< 0.001
50–60	230 (32.3)	165 (33.7)	48 (34.5)	17 (20.2)		23.5 (14.5)	
61–94	246 (34.5)	161 (32.9)	49 (35.3)	36 (42.9)		16.1 (9.6)	
Years since diagnosis							
< 1	360 (50.5)	243 (49.6)	66 (47.5)	51 (60.7)	0.273	25.9 (16.8)	0.802
1–2	263 (36.9)	181 (36.9)	55 (39.6)	27 (32.1)		25.9 (17.3)	
3–5	90 (12.6)	66 (13.5)	18 (12.9)	6 (7.1)		26.8 (18.7)	
Educational level							
Primary education or less	251 (35.2)	167 (34.1)	53 (38.1)	31 (36.5)	0.888	18.9 (12.9)	0.068
High school / vocational training	220 (30.8)	154 (31.4)	39 (28.1)	27 (31.8)		26.9 (16.4)	
University graduate	243 (34.0)	169 (34.5)	47 (33.8)	27 (31.8)		32.7 (19.1)	
Body mass index, kg/m^2^							
< 25	347 (49.2)	234 (48.3)	74 (53.6)	39 (46.4)	0.304	33.8 (18.5)	< 0.001
25–29.9	230 (32.6)	158 (32.6)	38 (27.5)	34 (40.5)		21 (12.7)	
≥ 30	129 (18.3)	92 (19.0)	26 (18.8)	11 (13.1)		14.7 (9.9)	
Energy intake, kcal/day							
< 1500	222 (31.7)	146 (30.2)	40 (29.9)	36 (43.4)	0.172	26.5 (18.5)	0.270
1500–1999	285 (40.7)	201 (41.6)	54 (40.3)	30 (36.1)		24.5 (16.5)	
≥ 2000	193 (27.6)	136 (28.2)	40 (29.9)	17 (20.5)		28.3 (16.9)	
Physical activity, min/wk							
< 150	490 (68.9)	335 (68.8)	95 (68.3)	60 (70.6)	0.765	25.6 (16.8)	0.209
150–300	97 (13.6)	68 (14.0)	16 (11.5)	13 (15.3)		25.6 (16.5)	
≥ 300	124 (17.4)	84 (17.2)	28 (20.1)	12 (14.1)		28.3 (19.6)	
Tobacco consumption							
No	555 (77.7)	383 (78.2)	104 (74.8)	68 (80.0)	0.611	24.5 (16.5)	0.436
Yes	159 (22.3)	107 (21.8)	35 (25.2)	17 (20.0)		31.6 (18.8)	
Alcohol consumption							
No	261 (36.6)	166 (33.9)	55 (39.6)	40 (47.1)	0.047	25.1 (18.1)	0.348
Yes	453 (63.4)	324 (66.1)	84 (60.4)	45 (52.9)		26.6 (16.8)	
Age at menarche							
< 12	192 (27.2)	135 (28.0)	34 (24.5)	23 (27.1)	0.709	22.4 (14.8)	0.003
≥ 12	514 (72.8)	347 (72.0)	105 (75.5)	62 (72.9)		27.5 (18.0)	
Age at first birth							
< 20	34 (4.8)	19 (3.9)	8 (5.8)	7 (8.2)	0.092	17.9 (12.0)	< 0.001
20–24	163 (22.9)	122 (24.9)	29 (20.9)	12 (14.1)		19.3 (13.8)	
25–29 or nulliparous	325 (45.6)	211 (43.1)	67 (48.2)	47 (55.3)		25.8 (16.6)	
≥ 30	191 (26.8)	137 (28.0)	35 (25.2)	19 (22.4)		33.7 (18.8)	
Contraceptives use							
Never	270 (37.8)	188 (38.4)	52 (37.4)	30 (35.3)	0.859	23.1 (16.6)	0.269
Past use	444 (62.2)	302 (61.6)	87 (62.6)	55 (64.7)		27.9 (17.5)	
Menopausal status							
Pre/perimenopausal	271 (38.0)	192 (39.2)	47 (33.8)	32 (37.6)	0.514	36.8 (17.8)	< 0.001
Postmenopausal	443 (62.0)	298 (60.8)	92 (66.2)	53 (62.4)		19.5 (13.2)	
Hormone replacement therapy use							
Never	642 (90.0)	443 (90.6)	127 (91.4)	72 (84.7)	0.208	26.8 (17.6)	0.417
Past use	71 (10.0)	46 (9.4)	12 (8.6)	13 (15.3)		19.1 (12.3)	
Family history of breast cancer							
None	426 (59.7)	294 (60.0)	82 (59.0)	50 (58.8)	0.659	26.4 (17.6)	0.672
Second degree only	156 (21.8)	102 (20.8)	36 (25.9)	18 (21.2)		26.5 (16.6)	
First degree	132 (18.5)	94 (19.2)	21 (15.1)	17 (20.0)		24.3 (17.1)	
Previous breast biopsies							
No	564 (79.0)	386 (78.8)	110 (79.1)	68 (80.0)	0.967	25.2 (16.6)	< 0.001
Yes	150 (21.0)	104 (21.2)	29 (20.9)	17 (20.0)		29.3 (19.3)	

Abbreviations: HR+  = hormone receptor positive tumors (estrogen receptor positive and/or progesterone receptor positive, with HER2 negative); HER2+  = human epidermal growth factor receptor 2 positive tumors; TN = triple negative tumors; SD: standard deviation

^a^ANOVA F-test for heterogeneity of mammographic density across categories of each characteristic, adjusted for age and body mass index. A separate model was fitted for each variable

^b^In tertiles

Table 2 shows the SPs of pathological BC subtypes by category of MD. Although no results were statistically significant, we observed that that women with MD ≥ 50% had lower SP of HR+ tumors (SPR = 0.87; 95% CI 0.67–1.13) and higher SP of HER2+ (SPR = 1.36; 95% CI 0.72–2.58) and TN tumors (SPR = 1.23; 95% CI0.47–3.22) compared to women with MD < 10%. We also observed that women with a MD between 25 and 50% had a higher prevalence of TN tumors (SPR = 1.31; 95% CI 0.66–2.60) than women whose MD was < 10%.

**Table 2:** Standardized prevalences of pathological tumor subtypes by category of mammographic density at diagnosis among women with breast cancer

		HR+			HER2+			TN		
Mammographic density	No. of women	No. of cases	Standardized prevalence^a^ (%; 95% CI)	Standardized prevalence ratio^a^ (95% CI)	No. of cases	Standardized prevalence^a^ (%; 95% CI)	Standardized prevalence ratio^a^ (95% CI)	No. of cases	Standardized prevalence^a^ (%; 95% CI)	Standardized prevalence ratio^a^ (95% CI)
< 10%	126	87	70.6 (61.4–78.3)	1.00 (reference)	26	19.5 (13.1–28.0)	1.00 (reference)	13	9.9 (5.6–16.9)	1.00 (reference)
10%– < 25%	251	173	69.5 (63.4–75.0)	0.98 (0.86–1.13)	48	18.7 (14.2–24.2)	0.96 (0.62–1.49)	30	11.8 (8.3–16.4)	1.18 (0.63–2.21)
25%– < 50%	250	172	67.7 (61.3–73.5)	0.96 (0.82–1.13)	46	19.3 (14.5–25.1)	0.99 (0.60–1.63)	32	13.0 (9.2–18.1)	1.31 (0.66–2.60)
≥ 50%	69	43	61.3 (47.9–73.1)	0.87 (0.67–1.13)	18	26.5 (16.4–39.9)	1.36 (0.72–2.58)	8	12.3 (5.9–23.7)	1.23 (0.47–3.22)

Abbreviations: HR+  = hormone receptor positive tumors (estrogen receptor positive and/or progesterone receptor positive, with HER2 negative); HER2+  = human epidermal growth factor receptor 2 positive tumors; TN = triple negative tumors; CI: confidence Interval

^a^Standardized to the overall distribution of age, recruiting region, body mass index, educational level, age at menarche, age at first birth, menopausal status, alcohol consumption and previous breast biopsies in the entire sample of women with breast cancer. Results based on 696 women with complete information

The stratified analyses by menopausal status and BMI (Table 3) with MD grouped as < 25% and ≥ 25%, showed no significant differences in the SPR. However, a higher SP of HER2+ and TN tumors in women with higher MD was only observed among pre/perimenopausal (HER2+ : SPR_MD≥25% vs MD<25%_ = 1.35; 95% CI 0.67–2.70 and TN: SPR_MD≥25% vs MD <25%=_1.30; 95% CI 0.58–2.90) and in those with BMI ≥ 25 kg/m^2^ (HER2+ : SPR_MD≥25% vs MD <25%_ = 1.22; 95% CI 0.73–2.02 and TN: SPR_MD≥25% vs MD <25%_ = 1.34; 95% CI 0.74–2.44).

**Table 3:** Standardized prevalences of pathological tumor subtypes by category of mammographic density at diagnosis in strata of menopausal status and body mass index at diagnosis of women with breast cancer

		HR+			HER2+			TN		
Mammographic density	No. of women	No. of cases	Standardized prevalence^a^ (%; 95% CI)	Standardized prevalence ratio^a^ (95% CI)	No. of cases	Standardized prevalence^a^ (%; 95% CI)	Standardized prevalence ratio^a^ (95% CI)	No. of cases	Standardized prevalence^a^ (%; 95% CI)	Standardized prevalence ratio^a^ (95% CI)
Pre/perimenopausal										
MD < 25%	67	51	76.7 (64.4–85.7)	1.00 (reference)	9	13.5 (6.9–24.6)	1.00 (reference)	7	9.8 (4.5–20.2)	1.00 (reference)
MD ≥ 25%	200	137	69.1 (60.0–76.9)	0.90 (0.76–1.07)	38	18.1 (12.2–26.2)	1.35 (0.67–2.70)	25	12.7 (7.6–20.5)	1.30 (0.58–2.90)
Postmenopausal										
MD < 25%	310	209	66.5 (59.4–72.9)	1.00 (reference)	65	21.9 (16.4–28.5)	1.00 (reference)	36	11.7 (7.8–17.1)	1.00 (reference)
MD ≥ 25%	119	78	66.4 (57.2–74.6)	1.00 (0.85–1.17)	26	21.1 (14.5–29.7)	0.97 (0.62–1.49)	15	12.4 (7.5–20.0)	1.07 (0.58–1.94)
*P* for homogeneity^b^				0.36			0.42			0.70
Body mass index < 25 kg/m^2^										
MD < 25%	121	79	66.1 (57.1–74.0)	1.00 (reference)	29	23.1 (16.4–31.4)	1.00 (reference)	13	10.9 (6.4–17.9)	1.00 (reference)
MD ≥ 25%	223	153	67.3 (60.1–73.8)	1.02 (0.86–1.21)	45	21.6 (16.1–28.4)	0.94 (0.60–1.47)	25	11.1 (7.4–16.4)	1.02 (0.52–2.00)
Body mass index ≥ 25 kg/m^2^										
MD < 25%	256	181	71.6 (65.4–77.0)	1.00 (reference)	45	16.7 (12.5–22.0)	1.00 (reference)	30	11.7 (8.1–16.6)	1.00 (reference)
MD ≥ 25%	96	62	64.0 (53.8–73.0)	0.89 (0.75–1.06)	19	20.3 (13.3–29.8)	1.22 (0.73–2.02)	15	15.7 (9.7–24.5)	1.34 (0.74–2.44)
*P* for homogeneity^b^				0.27			0.42			0.53

Abbreviations: HR+  = hormone receptor positive tumors (estrogen receptor positive and/or progesterone receptor positive, with HER2 negative); HER2+  = human epidermal growth factor receptor 2 positive tumors; TN = triple negative tumors; CI: confidence Interval; MD = mammographic density

^a^Standardized to the overall distribution of age, recruiting region, educational level, age at menarche, age at first birth, alcohol consumption, previous breast biopsies, and menopausal status and body mass index in analyses stratified by body mass index and menopausal status, respectively, in the entire sample of women with breast cancer. Results based on 696 women with complete information

^b^*P* for homogeneity of standardized prevalence ratios across strata of menopausal status and body mass index at diagnosis

**Fig. 1:**
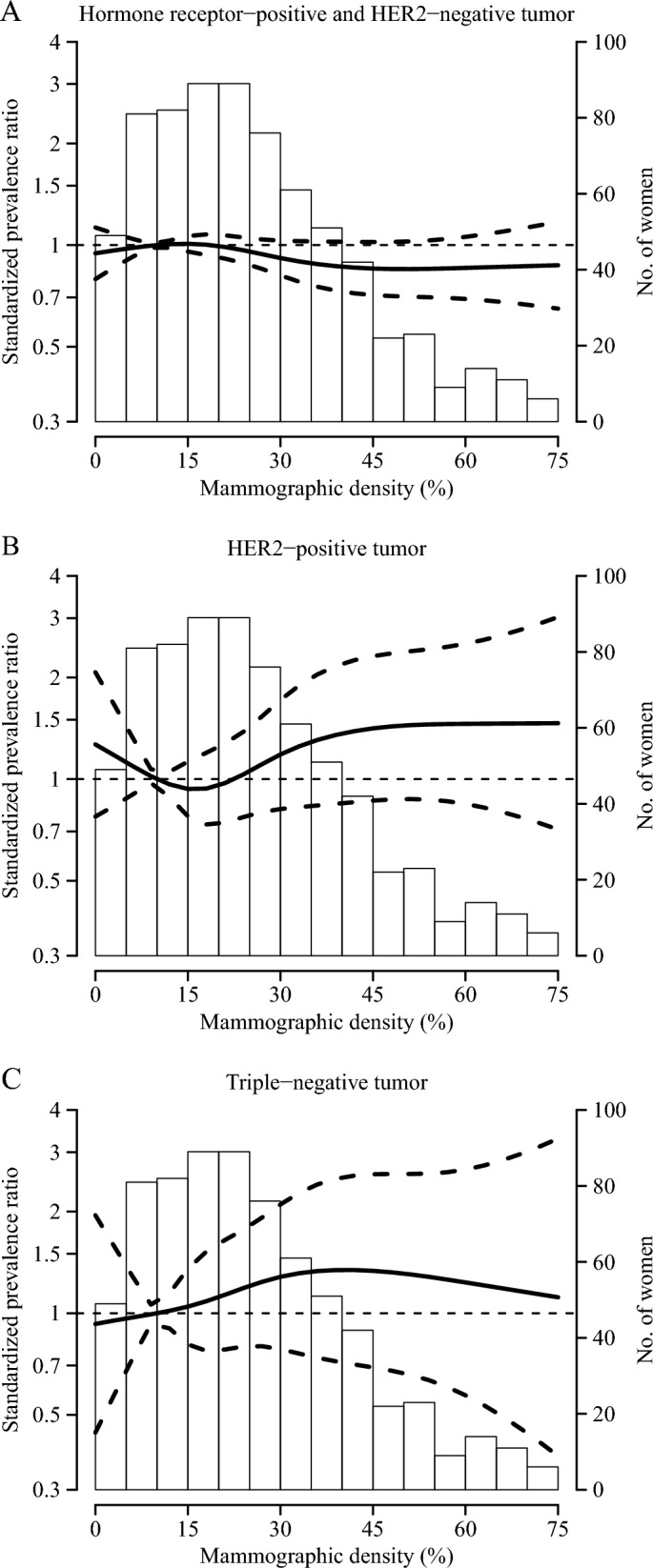
Standardized prevalence ratios for hormone receptor-positive and HER2-negative (**A**), HER2-positive (**B**), and triple-negative tumors (**C**) as a smooth function of mammographic density at diagnosis among women with breast cancer. Curves represent standardized prevalence ratios (solid lines) and their 95% confidence intervals (dashed lines) obtained from a multinomial logistic regression model based on restricted cubic splines for mammographic density with two internal knots at the 35th and 65th percentiles and boundary knots at the 5th and 95th percentiles. The reference value (prevalence ratio = 1) was set at 10% mammographic density. Prevalence ratios were standardized to the overall distribution of age, recruiting region, body mass index, educational level, age at menarche, age at first birth, menopausal status, alcohol consumption and previous breast biopsies in the entire sample of women with breast cancer. Results based on 696 women with complete information. Histogram represents the distribution of mammographic density

Figure 1 depicts the SPRs and their 95%CIs for the three pathological BC subtypes as a smoothed function of MD. The SPR of HR+ tumors appears to decrease with increasing MD, stabilizing around 45%. For HER2+ tumors, the SPR appears to increase between densities of 25% and 45%, and then stabilizes (with SP approximately 35–40% higher than in women with a 10% MD). Finally, the SPR of TN tumors shows an increasing trend with MD up to a density of about 40%, followed by a decrease, although the uncertainty is high due to the small number of TN tumor cases.

## Discussion

This study examines the association between MD and pathological BC subtypes, and assesses whether BMI and menopausal status modify this relationship. Overall, our results, although not statistically significant, suggest that the prevalence of HER2+ and TN tumors in pre/perimenopausal women and in those with BMI ≥ 25 kg/m^2^ might be higher among women with high MD.

There is ongoing debate about the relationship between MD and histological subtypes of BC. Evidence suggests that MD is a potent risk factor for most BC subtypes, although the extent of its influence on each subtype is controversial [15]. A review by Shawky et al., concluded that MD increases BC risk regardless of hormone receptor status or molecular subtype [13]. However, Bai et al., in a meta-analysis including five cohort/case–control and 18 case-only studies, found that increased MD is more strongly linked to HER2+ cancers compared to other BC subtypes (relative risk ratios for BI-RADS 4 versus 1 of 2.58 (95% CI 1.63–4.08), when compared HER2+ over TN tumors for case only studies) [15]. Our findings, indicate a non-significant higher prevalence of HER2+ tumors in women with high MD, aligning with the previously cited meta-analysis and with other studies [16, 17, 19, 27]. Regarding the positive association detected between MD and prevalence of TN tumors, a recent meta-analysis showed that higher MD was significantly associated with increased risk for TN BC (odds ratio = 2.19, 95% CI = 1.67–2.88) [28]. Three previous case–control studies [29–31] also found higher odds ratios for this type of tumor, although the differences between pathological subtypes were not statistically significant. The positive association observed only with HER2+ and TN tumors could be explained in part by the high proportion of stroma, containing fibroblasts, collagen and immune cells, which suggest a pro-tumor inflammatory microenvironment. This microenvironment can promote cell proliferation, even in the absence of hormonal influence, and could potentially lead to the growth of more aggressive non-hormone-dependent tumors. Complex interactions between the epithelium, stroma, and the extracellular matrix may also lead to DNA damage and mutations, and promote certain BC subtypes [32, 33]. The STAT signaling pathway could be involved in the stronger association detected with HER2+ tumors, since higher tumor pSTAT3 expression has been observed in patients with higher MD and in patients with HER2+ breast tumors [34]. In a pooled analysis of six studies, Bertrand et al. suggested that the positive association observed between percent MD and ER- disease among women < 55 years was driven by the inverse association of non-dense area with ER- disease, rather than by a positive association with absolute dense breast area [35]. On the other hand, dense breasts have been linked to reduced levels of terminal duct lobular unit (TDLU) involution in benign diagnostic breast biopsies [36], and to the absence of lobular involution in a cohort of women with pathologically confirmed benign breast disease [37]. Moreover, TDLU involution has been reported to be less marked in benign tissues adjacent to core basal BC than in the luminal A phenotype [38]. Taken together, these findings suggest that the greater prevalence of TN tumors among women with higher MD in our study may be partly explained by reduced breast tissue involution. In any case, and given that our results are based on a small number of cases, larger studies are needed for confirmation.

Although no statistically significant differences were observed when stratified by BMI and menopausal status categories, our analysis revealed that the positive association between MD and the prevalence of HER2+ and TN tumors was only observed in pre/perimenopausal women and in those with BMI ≥ 25 kg/m^2^. Regarding BMI, our findings contradicts the conclusions of Li et al. [16] and Tian et al. [17], who reported a stronger association between HER2+ tumors and MD among women with normal BMI. It is important to note, however, that both studies were conducted in Chinese women, with a low proportion of patients with fatty breast tissue, which may have limited the power to detect associations with MD. On the other hand, although Kleinstein’s research found no statistically significant differences in the association between MD and tumor subtype by BMI, the estimates were slightly stronger for HER2+ subtype among those with BMI ≥ 25 kg/m^2^ [19]. In another multicenter cross-sectional study carried out in the Spanish population, Calvo et al. also found no significant differences in the analysis of BC molecular subtypes and mammographic breast density according to three BMI groups [39]. Regarding menopausal status, our results are consistent with those obtained by McCarthy et al., who reported an association between higher MD and TN tumors, with a greater effect among premenopausal women [20]. Kleinstein et al. also detected stronger associations between percent MD and the TN subtype among younger women [19]. The more proliferative and proinflammatory environment of breast tissue in both premenopausal and overweight/obese women, could provide a favorable environment for the high density-related risk of HER2+ and TN tumors.

This study has several limitations that should be considered when interpreting our findings. First, the cross-sectional design did not allow us to determine temporal associations between MD and risk of specific tumor subtypes. Second, the multicenter hospital-based, case–case design may have introduced selection bias, potentially leading to an overrepresentation of certain tumor subtypes compared with their distribution in the general population. On the other hand, survivorship bias may also be present, as patients with poorer prognosis or more compromised health may have been less likely to participate. The study relied on self-reported data and, although more than half of the patients were diagnosed during the year prior to the interview, we cannot rule out a possible recall bias in our results. This bias could be more relevant in the group of women recruited retrospectively, in whom the mean time between diagnosis and interview was longer. Furthermore, the fact that participants answered the epidemiological questionnaire already knowing their pathology results may have influenced the way they reported their lifestyle habits and medical history, which could have affected the accuracy of the information collected. However, this influence would probably be non-differential among tumor subtypes. While this bias does not directly affect either the outcome or the exposure (MD), since the contralateral mammogram was collected at diagnosis before starting any treatment, it may still impact the measurement of covariates and, consequently, the control of confounding. Furthermore, the relatively small number of HER2+ cases—and especially of TN cases—limited our capacity to stratify the analyses by BMI and menopausal status. Consequently, our findings should be considered with caution, as the robustness of the conclusions may be affected. Additional studies with larger samples are needed to confirm the observed trend in the associations. Finally, although this is a multicenter study carried out in four Spanish regions, our sample is not representative of all BC cases in Spain.

Despite these limitations, the study has several strengths. We estimated prevalences and prevalence ratios controlling for a wide range of potential confounders by direct standardization to their distribution in the total sample, providing more comparable and consistent results. In addition, we evaluated possible “dose–response” associations, and interactions with BMI and menopausal status, in an attempt to clarify some of the inconsistencies detected in previous studies. Finally*,* MD was measured on a continuous scale, using a validated computer-assisted method and by a single reader, which showed high internal consistency. However, intra-reader drift over time cannot be completely ruled out in the absence of repeated calibration.

## Conclusions

Our findings suggest that increased MD could be associated with the development of more aggressive and non-hormone-dependent tumors, such as HER2+ and TN BC, particularly among pre/perimenopausal and overweight/obese women. These results should be interpreted with caution, as they are exploratory and limited by the cross-sectional, case–case design. Further studies are warranted to investigate potential biological mechanisms that could potentially link increased MD with specific tumor subtypes.

## Data Availability

The datasets used during the current study are available from the corresponding author upon reasonable request. The data are not publicly available due to restrictions imposed by the Ethics Committees of the participating hospitals.
