# Correction: Mammographic density and breast cancer pathological subtypes by menopausal status and body mass index

**DOI:** 10.1186/s13058-025-02169-5

**Published:** 2025-11-12

**Authors:** Julia Fernández-Morata, Marina Pollán, Nerea Fernández de Larrea-Baz, Vanessa Pachón-Olmos, Javier García-Pérez, Adela Castelló, María Ángeles Sierra, Pilar Lucas, Rafael Llobet, Agostina Stradella, Blanca Cantos, Teresa Ramón y Cajal, Marta Santisteban, Miguel Ángel Seguí, Ana Santaballa Bertrán, Mónica Granja, Julia Camps-Herrero, Sabela Recalde, Beatriz Nuñez-García, Nuria Calvo Verges, Beatriz Pérez-Gómez, Roberto Pastor-Barriuso, Virginia Lope

**Affiliations:** 1 Programa de Doctorado en Ciencias Biomédicas y Salud Pública, Instituto Mixto de Investigación Escuela Nacional de Sanidad UNED-IMIENS, Madrid, Spain; 2 Cancer and Environmental Epidemiology Unit, Department of Epidemiology of Chronic Diseases, National Center for Epidemiology, Instituto de Salud Carlos III (ISCIII), Madrid, Spain; 3 Consortium for Biomedical Research in Epidemiology and Public Health (CIBERESP), Madrid, Spain; 4 Servicio de Medicina Preventiva, Hospital Universitario La Paz-Carlos III-Cantoblanco, Madrid, Spain; 5 Institute of Computer Technology, Universitat Politècnica de València, Valencia, Spain; 6 Department of Medical Oncology, Multidisciplinary Breast Cancer Unit, Institut Català d’Oncologia, Idibell, Barcelona Spain; 7 Department of Medical Oncology, Hospital Universitario Puerta de Hierro, Majadahonda, Madrid Spain; 8 Department of Medical Oncology, Hospital de La Santa Creu I Sant Pau, Barcelona, Spain; 9 Department of Medical Oncology, Breast Cancer Unit, Clínica Universidad de Navarra, Pamplona, Spain; 10 IdiSNA, Navarra Institute for Health Research, Pamplona, Spain; 11 Department of Medical Oncology, Consorci Corporació Sanitaria Parc Tauli. Institut d’Investigació i Innovació Parc Taulí (I3PT-CERCA), Universitat Autònoma de Barcelona, Barcelona, Spain; 12 Medical Oncology Department, La Fe Health Research Institute (IISLaFe), La Fe University Hospital, Valencia, Spain; 13 Department of Medical Oncology, Hospital Universitario Clínico San Carlos, Madrid, Spain; 14 Radiology at Breast Health, Ribera Salud Hospitals, Valencia, Spain; 15 Department of Medical Oncology, Multidisciplinary Breast Cancer Unit, Institut Català d’Oncologia-Hospitalet, Barcelona, Spain

**Correction to: Breast Cancer Research (2025) 27:187** 10.1186/s13058-025-02142-2

Following the publication of the original article, the authors reported that the name Nerea Fernández de Larrea-Baz had been misspelled as Nerea Fernández de Larrea-Baza-Baz. The correct name is now reflected in this correction, and the original article has been updated accordingly.

